# Engagement of people with lived and living experience in the editorial process: reflections on the special series on the unregulated drug toxicity crisis in Canada

**DOI:** 10.24095/hpcdp.44.7/8.01

**Published:** 2024-07

**Authors:** Pam Young, Charlene Burmeister, Amanda Slaunwhite, Heather Palis

**Affiliations:** 1 Unlocking the Gates Services Society, Maple Ridge, British Columbia, Canada; 2 Coalition of Substance Users of the North (CSUN), Quesnel, British Columbia, Canada; 3 School of Population and Public Health, University of British Columbia, Vancouver, British Columbia, Canada

## Introduction

Unregulated drug toxicity deaths (or “overdoses” or “poisonings”) remain an ongoing national public health emergency in Canada.^1^ Based on the available evidence, it is increasingly recognized that these deaths are a direct result of the criminalization of substance use, the failed war on drugs and outdated drug policy.^2^ Coroner records are an important source of data on unregulated drug toxicity deaths, providing information about the circumstances of death (e.g. location of death, contact with health care prior to death, postmortem toxicology, etc.). Each province and territory has its own approach to collecting data, and these have not been previously gathered to examine national unregulated drug toxicity events. This special series includes five articles, each focusing on a specific topic, using data from across all provinces and territories, to provide a national picture of unregulated drug toxicity events in Canada.^3^-^7^

## Engaging people with lived or living experience in research

This series had Guest Editors, including two people with lived or living experience (PWLLE) of substance use and two researchers. This is the first time that *Health Promotion and Chronic Disease Prevention in Canada* has engaged PWLLE in the editorial process.

Historically, PWLLE have not been effectively engaged in research.^8^ PWLLE have reported experiencing stigma; facing power imbalances, disrespect, inequality, challenges in accessing meeting materials and lack of flexibility; and experiencing a lack of trauma-informed approaches.^8^ Given this history, there is a need for meaningful engagement of PWLLE across the research process—from designing research questions, and collecting and interpreting data based on their lived experiences, to writing, presenting at conferences, peer review and engagement in editorial roles.

It is critical to work toward inclusive priority-setting in research to seek to respond to the drug toxicity crisis. This can be achieved by building partnerships between researchers and PWLLE that centre the expertise of PWLLE. Engagement of PWLLE from the inception of research is critical so that research questions reflect priorities for data collection that respond to the needs of communities. Such engagement can ensure that research findings are used in a manner that is helpful rather than harmful, for example, avoiding misinterpreting data used to inform priority setting (i.e. resource allocation, service provision, policy change) to address the current crisis.

Researchers working with PWLLE of substance use need to consider the ongoing harms of criminalization.^9^,^10^ The Guest Editors highlighted that authentic and meaningful engagement of PWLLE is necessary to move toward emancipating people who use drugs. In this editorial, we report on the reflections of the Guest Editors on the contents of this special issue and the engagement process, taking this history into account.

## Overview of the special series content: reflections and recommendations

This special series examined data from across Canada, and this issue reports more specifically on the unregulated drug toxicity crisis as it relates to chronic pain,^5^ housing,^6^ and impacts across life stages.^7^ This series provides a first step toward a national picture of the circumstances of deaths, which has been an ongoing gap in the literature.

Nevertheless, there are important limitations. First, the data are from 2016. While this provides some historical context, there are significant limitations to the generalizability of the findings to the present day. Moreover, given that data were abstracted from coroner records from settings with different reporting structures and protocols, data relevant to the variables of interest were available only in some regions. The high proportion of missing data in some analyses limited the ability to reach conclusions.

To address these limitations, there is a need for more timely data and coordination of responses to allow these data to be accessed in near real-time, as was seen with the COVID-19 public health response.^11^ Collection methods across regions need to be coordinated to support standardized reporting. This will allow for more meaningful comparisons, which are currently lacking.

The studies in this special series provide a historical snapshot of the unregulated drug toxicity crisis in Canada. Data can be interpreted relative to what we know today, representing a journey over time and showing changes in the unregulated toxic drug supply, expansion of drug user organizations, and increased availability of treatment and harm reduction services. While the implementation of harm reduction and treatment services has prevented deaths,^12^ incremental efforts toward service provision have been insufficient to curb the ongoing unregulated drug toxicity crisis, which continues to be a public health emergency in 2024.

## Overview of engagement of PWLLE as Guest Editors: reflections and recommendations

In the context of engaging front-line workers in academic activities, peer overdose response workers have emphasized the importance of their work being recognized (e.g. through financial compensation, co-authorship), organizational support and skills development,^13^,^14^ all of which we prioritized in the process of engaging PWLLE as Guest Editors for this special series.

This engagement was facilitated through regular meetings of the researcher and PWLLE Guest Editors. Information was made available to the PWLLE Guest Editors ahead of these meetings; however, meetings did not begin with the expectation that they had reviewed the documents. Documents were reviewed together, to be sure everyone was starting from the same place. This was critical for reasons that are relevant to engaging PWLLE in future editorial roles: workload outside of editorial duties, learning-accommodation requirements, vicarious trauma and traumatic personal life experiences, and the many commitments PWLLE have outside of these meetings. The dedicated group meeting time was organized to suit the PWLLE Guest Editors’ approach to the work, acknowledging the competing priorities in their community-based peer-led work.

The engagement with the Guest Editors was mutually beneficial. The PWLLE Guest Editors described this as a valuable experience, as they learned new academic language and research methods and gained confidence for engaging in similar activities in the future. In turn, the researcher Guest Editors were challenged to ask new questions about the data and bring a new lens to the review based on their PWLLE colleagues’ input.

Engaging two peers was critical to alleviating the burden on one person. The PWLLE Guest Editors bounced ideas off one another, affirming or challenging one another’s perspectives, which ultimately helped to move the discussion toward how to best revise each manuscript.

A key learning was that engaging PWLLE Guest Editors is a time-intensive process. Future engagement of PWLLE in Guest Editor roles across academic disciplines and journals should make deadlines more flexible to acknowledge the time needed for professional development and to develop new processes and protocols for engagement.

Including PWLLE as Guest Editors required significant time and thought as it was a new practice for the journal. For this special series, the PWLLE Guest Editors provided their expertise to ensure the included manuscripts were interpreted relative to the current real-world context of the unregulated drug toxicity crisis in Canada.

While guidelines exist for the engagement of PWLLE in the grant proposal and review process,^15^ to our knowledge, such guidance does not exist in terms of PWLLE as Guest Editors in research and knowledge translation. The engagement of PWLLE as Guest Editors for this special series has led to extremely valuable insights that could serve as a foundation for developing such guidelines. This engagement is a process that could be replicated by other journals, potentially strengthening the meaning and impact of academic research across the field of public health.

## Statement

The content and views expressed in this article are those of the authors and do not necessarily reflect those of the Government of Canada.
